# Iliac Crest Bone Grafting for the Management of Anterior Shoulder Instability in Patients with Glenoid Bone Loss: a Systematic Review of Contemporary Literature

**DOI:** 10.1186/s40798-020-0240-x

**Published:** 2020-02-11

**Authors:** Michael-Alexander Malahias, Dimitrios Chytas, Vasileios Raoulis, Efstathios Chronopoulos, Emmanouil Brilakis, Emmanouil Antonogiannakis

**Affiliations:** 1 3rd Orthopaedic Department, Hygeia Hospital, Erythrou Stavrou 4, Marousi, 15123 Athens, Greece; 2 2nd Orthopaedic Department, School of Medicine, National & Kapodistrian University of Athens, Agias Olgas 3, Nea Ionia, 14233 Athens, Greece; 3 Department of Orthopaedic Surgery, Faculty of Medicine, School of Health Sciences, University of Thessaly, Larissa, Greece

**Keywords:** Glenoid bone block, Anterior shoulder instability, Anterior shoulder dislocation, Glenoid bone loss, Iliac crest bone graft

## Abstract

**Background:**

A number of clinical trials have been published assessing the role of iliac crest bone grafting for the management of recurrent anterior instability with glenoid bone loss in contemporary practice. We therefore performed a systematic review of contemporary literature to examine the effect of iliac crest bone grafting on postoperative outcomes of these patients. Our hypothesis is that contemporary iliac crest bone block techniques are associated with low reoperation and complication rates combined with satisfactory functional results.

**Methods:**

The US National Library of Medicine (PubMed/MEDLINE), the Cochrane Database of Systematic Reviews, and EMBASE were searched between January 2008 and December 2019 for relevant publications.

**Results:**

Following the application of the inclusion-exclusion criteria, nine articles were found eligible for our analysis. In total, 261 patients (mean age range, 25.5–37.5 years; mean follow-up range, 20.6–42 months) were included in the studies of the current review. The mean modified Coleman score was 48.6 (range 37–65), indicating an overall low-to-moderate methodological quality. In the short term, the overall all-cause reoperation rate was 6.1%, while the rate of recurrent instability was 4.8%. The graft non-union rate was 2.2%, while the rate of osteolysis, graft fracture, and infection was 0.4%, 0.9%, and 1.7%, respectively. Finally, hardware-related complications, such as screw breakage or symptomatic mechanical irritation around the screw insertion, occurred in 3.9% of the patients.

**Conclusions:**

Iliac crest bone block techniques in contemporary practice are safe and effective in the short-term (< 4 years) follow-up for the management of anterior shoulder instability with substantial glenoid bone deficiency. However, further studies of higher quality and longer follow-up are required to establish the therapeutic value of these techniques as well as to clarify whether there are differences in the outcomes of arthroscopic and open iliac crest bone block procedures.

## Key Points

- Iliac crest bone block techniques in contemporary practice might be safe and effective for the management of anterior shoulder instability with glenoid bone deficiency.
- Further studies of higher quality and longer follow-up are required to establish the therapeutic value of iliac crest bone block techniques in contemporary practice.

## Background

Previous clinical and biomechanical studies have illustrated the importance of intact glenoid anatomy for shoulder stability [1, 2]. In cases with substantial anterior-inferior glenoid osseous defects, isolated soft tissue repair techniques have been shown inadequate in restoring shoulder stability, since they have been related to high postoperative recurrence rates of up to 40% [3–7]. It is generally believed that cases with large glenoid bone defects would require a bone block augmentation technique to restore joint stability [8, 9]. The Latarjet procedure with its various amendments has been the most commonly used bone block technique [10, 11]. However, while the Latarjet procedure has proved to be reliable to manage recurrent anterior shoulder instability with large glenoid bone defects, there have been concerns of a high surgical complication rate associated with this procedure [12–14]. A large recent review reported an overall complication rate in the open Latarjet procedure of 15%, with a 7% rate of unplanned reoperations [15].

An alternative option that has been used either for the revision of failed Latarjet procedures [16] or for the primary treatment of glenoid bone loss [10] is the Eden-Hybinnette technique. The initial procedure which has a 100-year history was based on the concepts of anatomic glenoid bony augmentation with a tibial autograft and capsulorrhaphy [17]. The traditional glenoid reconstruction has historically been associated with increased risk of postoperative degenerative changes [18].

Recently, several modifications in regard to the surgical approach, graft positioning and fixation, and the origin of the graft have been introduced [17]. In contrast to the modern techniques, in the original technique, Eden and Hybbinette placed the bone block inside the capsule at the anterior glenoid without any fixation and not flush with the glenoid, but as a mechanical dislocation barrier (not anatomically). Contemporary Eden-Hybinnette technique can be described by the use of an iliac crest bone block fixed with “low-profile” implants (buttons, sutures, J-shaped implant-free bone graft) through minimized open or arthroscopic procedures. A number of clinical trials have been published assessing the role of iliac crest bone grafting for the management of recurrent anterior instability with glenoid bone loss in contemporary practice. We therefore performed a systematic review of contemporary literature to examine the effect of iliac crest bone grafting technique on postoperative outcomes of these patients. Specifically, we aimed to answer the following questions: (1) what are the clinical and functional outcomes of the iliac crest bone block technique for the treatment of anterior shoulder instability with glenoid bone loss in contemporary practice? (2) Do contemporary iliac crest bone block techniques result in adequate bone graft healing, union, and osseous incorporation? (3) Do arthroscopic iliac crest bone block techniques result in similar clinical and radiographic outcomes compared to the respective open techniques? Our hypothesis is that contemporary iliac crest bone block techniques are associated with low reoperation and complication rates combined with satisfactory functional results.

## Methods

### Search Criteria

**Fig. 1:**
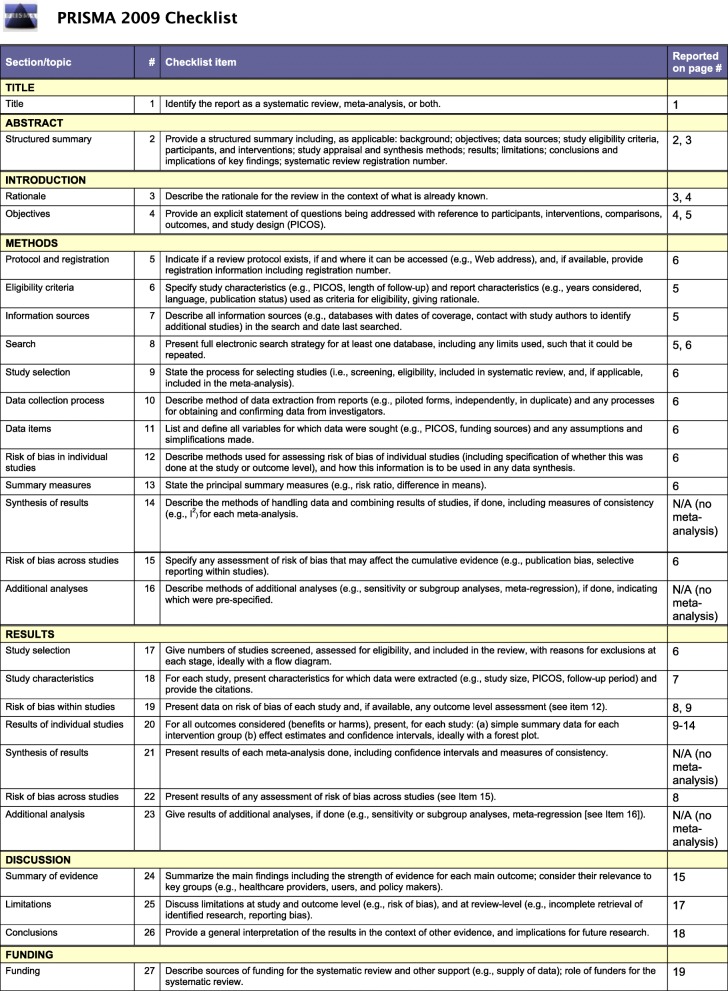
PRISMA 2009 checklist

The US National Library of Medicine (PubMed/MEDLINE), Cochrane Database of Systematic Reviews, and EMBASE were searched (from December 2007 to December 2019) according to the Preferred Reporting Items for Systematic Reviews and Meta-Analyses (PRISMA) (Fig. 1) for publications utilizing keywords pertinent to anterior shoulder instability, glenoid bone loss, iliac crest bone graft, and clinical outcomes. Only abstracts that evaluated the utility of iliac crest bone grafting in anterior shoulder instability with glenoid bone loss were reviewed. The specific search terms are further shown in Table 1. To maximize the search, backward chaining of reference lists from retrieved papers was also undertaken. A preliminary assessment of only the titles and abstracts of the search results was initially performed. The second stage involved a careful review of the full-text publications.

**Table 1:** Search criteria used

PubMed	Cochrane	EMBASE
glenoid bone loss[tw] OR glenoid defects[tw] OR glenoid defect[tw] AND (Anterior[tw] AND "Shoulder dislocation"[Mesh]) OR Anterior shoulder instability[tw] OR Anterior shoulder dislocation[tw] OR Anteroinferior Shoulder Instability[tw] OR shoulder instability[tw] AND iliac crest bone grafting[tw] OR iliac crest bone graft[tw] OR iliac crest bone grafts[tw] OR iliac graft[tw] OR iliac grafts[tw] OR glenoid bone block[tw] OR Arthroscopic bone block grafting[tw] OR J-bone graft[tw]	((("glenoid bone loss":ti,ab,kw OR "glenoid defects":ti,ab,kw OR "glenoid defect":ti,ab,kw)) AND ((Anterior:ti,ab,kw AND [mh "Shoulder dislocation"]) OR "Anterior shoulder instability":ti,ab,kw OR "Anterior shoulder dislocation":ti,ab,kw OR "Anteroinferior Shoulder Instability":ti,ab,kw OR "shoulder instability":ti,ab,kw)) AND ("iliac crest bone grafting":ti,ab,kw OR "iliac crest bone graft":ti,ab,kw OR "iliac crest bone grafts":ti,ab,kw OR "iliac graft":ti,ab,kw OR "iliac grafts":ti,ab,kw OR "glenoid bone block":ti,ab,kw OR "Arthroscopic bone block grafting":ti,ab,kw OR "J-bone graft":ti,ab,kw)	((("glenoid bone loss":ti,ab,de,tn,kw OR "glenoid defects":ti,ab,de,tn,kw OR "glenoid defect":ti,ab,de,tn,kw)) AND ((Anterior:ti,ab,de,tn,kw AND 'Shoulder dislocation'/exp) OR "Anterior shoulder instability":ti,ab,de,tn,kw OR "Anterior shoulder dislocation":ti,ab,de,tn,kw OR "Anteroinferior Shoulder Instability":ti,ab,de,tn,kw OR "shoulder instability":ti,ab,de,tn,kw)) AND ("iliac crest bone grafting":ti,ab,de,tn,kw OR "iliac crest bone graft":ti,ab,de,tn,kw OR "iliac crest bone grafts":ti,ab,de,tn,kw OR "iliac graft":ti,ab,de,tn,kw OR "iliac grafts":ti,ab,de,tn,kw OR "glenoid bone block":ti,ab,de,tn,kw OR "Arthroscopic bone block grafting":ti,ab,de,tn,kw OR "J-bone graft":ti,ab,de,tn,kw)

### Inclusion and Exclusion Criteria

The inclusion criteria were (1) studies describing human subjects of any age and sex, (2) studies that include a population of > 5 patients who were originally treated with an iliac crest bone block technique for the reconstruction of glenoid bone insufficiency in patients with anterior shoulder instability, (3) studies that follow patients for a minimum of 18 months after surgery, and (4) studies that provide a clinical/functional and/or radiographic outcome measure (e.g., patient-reported outcome scores, postoperative complications, functional scores, range of motion, pain scale). The exclusion criteria were (1) review articles, (2) case studies with ≤ 5 patients, (3) technical notes, (4) corrigenda, (5) editorial notes, (6) non-full-text articles, (7) studies without any clinical/functional or radiographic outcome, (8) studies in which patients were treated with coracoid transfer or other than iliac crest types of bone graft or soft-tissue surgery, (9) studies in which no subjects underwent iliac crest bone block technique, (10) non-English language publications, (11) studies published before December 2, 2007, or after December 2, 2019, (12) studies including patients who were operated on before 2000, and (13) studies with follow-up < 18 months.

### Data Collection

Two authors independently conducted the search [MM, DC]. To maximize the search, backward chaining of reference lists from retrieved papers was also undertaken. A preliminary assessment of only the titles and abstracts of the search results was initially performed. The second stage involved a careful review of the full-text publications. All authors compiled a list of articles not excluded after application of the inclusion and exclusion criteria. Discrepancies between the authors were resolved by discussion. In cases of disagreement, the senior author (EA) had the final decision.

During initial review of the data, the following information was collected for each study: title, author, year published, study design, number of patients, number of operated joints, sex, type of interventional treatment performed, type of graft, type of implant used, success percentage of treatment (free from recurrence of instability), reoperation rate, complication rate, return to sports, range of motion, clinical/functional subjective scores, and progression of glenohumeral osteoarthritis.

The methodological quality of each study and the different types of detected bias were assessed independently by each reviewer [MM, DC] with the use of modified Coleman methodology score [19, 20]. Selective reporting bias like publication bias was not included in the assessment. The primary outcome measure was the free-from-recurrence-of-instability rate, and the clinical, functional, and radiographic outcomes. Secondary outcome was the quality assessment of the studies with the use of the modified Coleman methodology score.

## Results

**Fig. 2:**
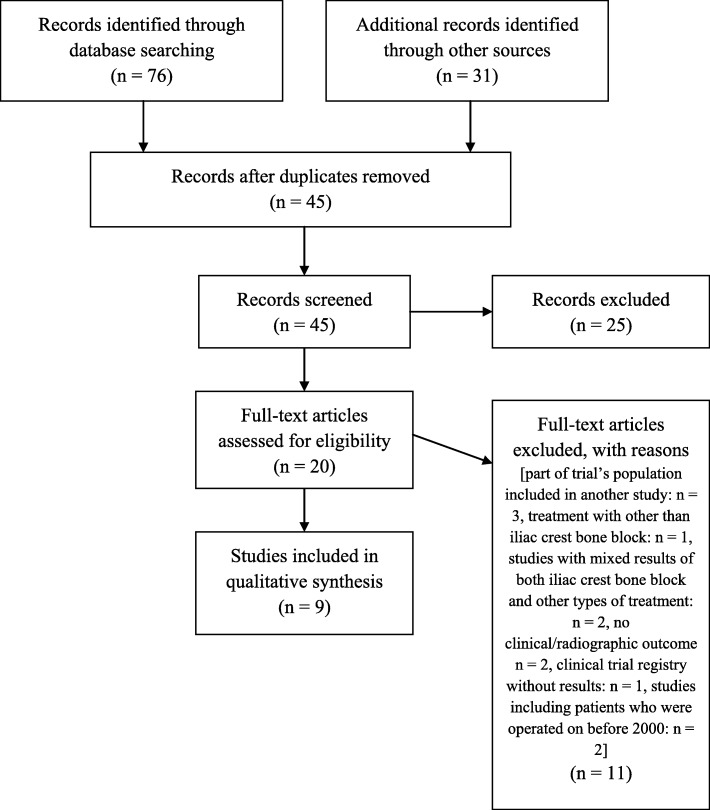
Flow chart of study selection according to PRISMA guidelines for reporting systematic reviews and meta-analyses

The literature search identified 76 abstracts that were examined to determine the efficacy of the iliac crest bone block technique for the management of glenoid bone loss in patients with anterior shoulder instability (Fig. 1). Following the application of the inclusion-exclusion criteria, nine articles were found eligible for our systematic analysis [21–28]. A summary flowchart of our literature search according to PRISMA guidelines can be found in Fig. 2.

### Demographics

Totally, 261 patients (217 males, 44 females) were included in the studies of the current review. From them, 231 patients underwent placement of iliac crest graft (40 with an open procedure, 191 with the arthroscopic procedure) and 30 patients underwent the Latarjet procedure. Their mean age ranged from 25.5 [21] to 37.5 years old [22]. The mean follow-up ranged from 20.6 months [23] to 42 months [25] (Table 2).

**Table 2:** Main characteristics of the studies of the current review

Study	Type	LOE	MCS	Risk of bias	COI	Number of patients	Mean age (y)	Sex	Mean FU (m)	Type of graft	Type of fixation	Mean clinical scores
Taverna et al. [21]	Retrospective Non-comparative	IV	56	Selection, detection, and performance	Yes	26	25.5	20M 6F	29.6	Iliac crest allograft	Two coupled pairs of arthroscopic round endobuttons	Walch-Duplay score: 93.2 (post-operatively) Rowe score: 96.4 (post-operatively) SSV: 87.4 (post-operatively)
Kraus et al. [23]	Retrospective Non-comparative	IV	39	Selection, detection, and performance	Yes	15	31.4	14M 1F	20.6	Autologous tricortical iliac crest bone graft	Two bio-compression screws	Constant score (affected side): 85.8 points Constant score (contra-lateral side): 89.6 points Rowe score: from 37.5 (pre-operatively) to 88.0 points (post-operatively) (significant difference) SSV: from 41.5 (pre-operatively) to 84.5% (post-operatively) (significant difference) WOSI: from 19.2% (pre-operatively) to 76.7% (post-operatively) (significant difference)
Anderl et al. [28]	Prospective Non-comparative	IV	53	Selection, detection, and performance	Yes	14 (15 shoulders)	30	12M 2F	25.9	Implant-free bi-cortical iliac crest bone grafting (autologous)		Rowe score: from 57.6 (pre-operatively) to 98.6 (post-operatively) (significant difference) Constant score from 70.9 (pre-operatively) to 96.3 (post-operatively) (significant difference) VAS score: 4.4 (pre-operatively) to 0.2 (post-operatively) (significant difference) SSV for sports: 31.4% (pre-operatively) to 95.6% (post-operatively) (significant difference)
Bock-mann et al. [25]	Retrospective Non-comparative	IV	40	Selection, detection, and performance	Not rep.	32	31	24M 7F	42	Iliac crest bone graft (autologous)	Two screws	WOSI: 71% Adapted constant score: 87 VAS score: 2.1
Zhao et al. [27]	Retrospective Non-comparative	IV	65	Selection, detection, and performance	No	52	26.3	39M 13F	38.8	Allogenic iliac bone graft	Sutures	Oxford shoulder score: from 29.7 pre-operatively to 42.4 post-operatively Rowe score: from 34.7 pre-operatively to 91.8 post-operatively
Gian-nakos et al. [22]	Retrospective Non-comparative	IV	43	Selection, detection, and performance	Yes	12	37.5	9M 3F	28.8	Re-vision setting, autologous bi-cortical iliac crest graft	2 cannulated titanium lag screws	Rowe score: from 16.25 points pre-operatively to 69.58 points post-operatively (significant difference) Walch-Duplay score: from 11.76 points pre-operatively to 76.67 points post-operatively (significant difference) WOSI (only post-operatively): 28.71%
Ernst-Brunner et al. [26]	Retrospective Comparative	III	37	Selection, detection, and performance	Yes	40 (20 open and 20 arthroscopic procedures)	30.9	35M 5F	–	Autologous iliac crest bone graft	–	No clinical outcomes.
Moroder et al. [24]	Prospective Randomized	I	57	–	No	60 (30 open Latarjet, 30 open iliac crest graft procedures)	31 (Latar-jet) 29 (iliac crest graft)	56M 4F	24	Autologous iliac crest bone graft	Two screws (Latarjet group) Implant-free press-fit insertion of J-shaped iliac crest bone graft	WOSI, Rowe, SSV, ASOSS score: insignificant difference between the 2 groups post-operatively (only preoperative Rowe score significantly better in Latarjet group)
Scheibel et al. [29]	Retrospective Non-comparative	IV	47	Selection, detection, and performance	No	10	28.7	8M 2F	37.9	Tri-cortical autogenous iliac crest bone graft	Two cannulated screws	Constant score: 88.3, Rowe score: 89.5, Walch-Duplay score: 83.5 MISS: 80.6, WOSI: 82.6% (only post-operative values)

### Level of evidence and quality of the studies

One out of nine studies of this review (11.1%) had a level of evidence III [26], one (11.1%) had a level of evidence I [24], while all other studies (77.8%) had a level of evidence IV (Table 2).

Apart from the study by Moroder et al. [24], all the other papers were characterized by selection, detection, and performance bias, while all apart from three studies [24, 27, 29] (33.3%) declared that some of their authors had a relevant conflict of interests. The mean modified Coleman methodology score was 48.6, while it ranged from 37 [26] to 65 [27] indicating an overall low-to-moderate methodological quality (Table 2).

### Type of Graft and Fixation

Seven studies (77.8%) described the use of autologous iliac crest grafts, whereas the remaining two studies (22.2%) made use of iliac crest allografts [21, 27]. Four studies (44.4%) used screws for the bone block fixation [22, 23, 25, 29], while one study (11.1%) used endobuttons [21] and the other one (11.1%) sutures [27]. Finally, there were two studies (22.2%) that examined the use of a J-shaped formed iliac crest bone block that fixed without any implants (implant-free technique) [26, 28] and one study [24] (11.1%) that comprised two groups: in the first one, the coracoid graft was fixed with screws, while in the second one, a J-shaped formed iliac crest bone block was fixed without any implants (Table 2).

### Clinical/functional subjective scores

Eight out of the nine studies (88.9%) reported clinical or functional subjective outcome variables [21–25, 27–29]. All mean postoperative clinical/functional subjective scores which were reported in the studies of this review were significantly improved compared to the respective mean preoperative values. The Rowe score was the most commonly used, since it was documented in seven studies (77.8 % of all studies) [21–24, 27–29]. The Western Ontario Shoulder Instability Index (WOSI) was used in five studies (55.6%) [22–25, 29]. The Constant score was utilized in four studies (44.4% of all studies) [23, 25, 28, 29] as well as the subjective shoulder value (SSV) [21, 23, 24, 28]. The Walch-Duplay score was measured in three studies (33.3%) [21, 22, 29]. The visual analog scale (VAS) score was measured in two studies (22.2% of all studies) [25, 28]. Finally, the Oxford Shoulder Score was examined in one study (11.1% of all) [27], the Athletic Shoulder Outcome Scoring System (ASOSS) in one study (11.1%) [24], and the Melbourne Instability Shoulder Score (MISS) in one study (11.1%) [29] (Table 2).

### Range of Motion

Seven out of the nine studies (77.8%) dealt with the range of motion (ROM) of their patients’ operated shoulder [22–25, 27–29]. Kraus et al. [23] did not find significant differences in any of the different elements of postoperative ROM compared to the healthy contralateral side. Scheibel et al. [29] found that the mean hand-to-back distance of the affected side (achieved actively during the lift-off test) was significantly inferior to that of the healthy side. In addition, Anderl et al. noted that all elements of ROM were significantly improved compared to the preoperative respective values [28]. Bockmann et al. reported that their patients achieved full ROM in the abduction and external rotation post-surgery [25], while Zhao et al. noted that almost all patients of their cohort had normal postoperative ROM [27].

### Clinical/Functional Subjective Scores

Eight out of the nine studies (88.9%) reported clinical or functional subjective outcome variables [21–25, 27–29]. All mean postoperative clinical/functional subjective scores which were reported in the studies of this review were significantly improved compared to the respective mean preoperative values. The Rowe score was the most commonly used, since it was documented in seven studies (77.8% of all studies) [21–24, 27–29]. The Western Ontario Shoulder Instability Index (WOSI) was used in five studies (55.6%) [22–25, 29]. The constant score was utilized in four studies (44.4% of all studies) [23, 25, 28, 29] as well as the SSV [21, 23, 24, 28]. The Walch-Duplay score was measured in three studies (33.3%) [21, 22, 29]. The VAS score was measured in two studies (22.2% of all studies) [25, 28]. Finally, the Oxford Shoulder Score was examined in one study (11.1% of all) [27], the Athletic Shoulder Outcome Scoring System (ASOSS) in one study (11.1%) [24], and the MISS in one study (11.1%) [29] (Table 2).

### Return to Athletic Activities

Three out of the nine studies (33.3%) documented return to sports after surgery as one of the success rate criteria [21, 22, 28]. Both Taverna et al. [21] and Giannakos et al. [22] found that two thirds of their patients who played sports before injury (66.7%) returned to their pre-injury level of athletic activity. In addition, Anderl et al. noted that all patients returned to their pre-injury level of athletic activity within 6 months after surgery [28].

### Complications

The overall all-cause reoperation rate was 6.1% (14 out of 231 patients), while the overall complication rate was 19.9% (46 out of 231 patients).

#### Recurrent Instability (Dislocation, Subluxation, Positive Apprehension Test)

There were 11 cases of recurrent dislocation or subluxation (out of 231 patients; 4.8%). Three of these 11 cases were re-operated, whereas the other eight cases were treated conservatively. In addition, there were 11 cases of persistent positive apprehension test (4.8% of all patients), one of which underwent plication of the capsule [23].

#### Osteolysis and Non-union of the Graft

In total, two out of the nine studies of the current review (22.2%) reported cases of non-union or osteolysis of the bone graft [21, 22]. Overall, there were five cases (out of 231 patients; 2.2%) of non-union and one case of osteolysis (0.4%).

#### Infection Rate

Two out of the nine studies (22.2%) reported in total four postoperative cases of infection [24, 25] (four patients out of 231, rate 1.7%).

#### Hardware-Related Complications

The rate of hardware-related complications was 3.9% (9 out of 231 cases). Bockmann et al. reported that two out of the 32 patients (6.3% of their patient cohort) experienced mechanical irritation around the screw insertion sites generating persistent pain [25]. These patients were successfully treated with arthroscopic removal of the screw [25]. Zhao et al. found two out of the 52 cases (3.8% of their patient cohort) with posterior-inferior penetration of the glenoid by the tip of the anchors [27]. In addition, Giannakos et al. noted that four out of the 12 patients (33.3% of their patient cohort) required hardware removal due to possible contact between humeral head and screws [22]. Moreover, one patient was radiographically diagnosed with screw breakage which did not require revision surgery [22].

#### Other Complications

Other complications, which were not previously described, were diagnosed in 16 patients (6.9% of all patients). Neurological hypoesthesia at the donor site was observed in 10 patients (out of 128 with iliac crest harvesting, 7.8%) and graft fracture in two cases (out of 231, 0.9%). Finally, one study reported postoperative hematoma in two out of the 26 patients (7.7%) which resolved spontaneously.

### Postoperative Progression of Osteoarthritis

Six out of the nine studies of the present review (66.7%) assessed the presence of glenohumeral osteoarthritis [21–23, 27–29]. In total, nine cases (out of 231, 3.9%) of the progression of glenohumeral osteoarthritis were noted.

### Arthroscopic Versus Open Iliac Crest Bone Block Technique

One out of the nine studies (11.1%) compared the radiographic outcome of arthroscopic versus open glenoid reconstruction with iliac crest bone block graft [26]. The covered defect size was significantly different amongst groups (95% in the arthroscopic group, 98% in the open group) [26]. The arthroscopic group showed a significantly steeper mean impaction angle (34.8°) and significantly increased mean medial offset (6.6 mm) compared to the open group (mean impaction angle of 26.9°, mean medial offset 5.4 mm) [26]. Finally, the mean difference in the mediolateral step formation amongst groups was not significant (2.9 mm in the arthroscopic group and 3.2 mm in the open group) [26].

### Iliac Crest Bone Block Versus Coracoid Transfer Technique

One out of the nine studies of the current review (11.1%) compared the outcomes of iliac crest bone graft (open) technique and open coracoid transfer technique (Latarjet) for the treatment of anterior shoulder instability with the glenoid bone loss [24]. Moroder et al. [24] did not find any significant differences in the failure rates of the Latarjet group and the iliac crest graft group. The two procedures did not differ significantly in WOSI, Rowe score, SSV, or ASOSS score at any follow-up time point, while internal rotation was significantly higher in the iliac crest graft group compared to the Latarjet group. Furthermore, there were not any significant differences between the two groups in postoperative pain, satisfaction, strength, abduction, and external rotation at the final follow-up. Finally, the defect area was significantly lower in the iliac crest graft group at final follow-up.

### Iliac Crest Bone Autograft Versus Iliac Crest Bone Allograft in Glenoid Reconstruction

No study was found to compare the outcomes of iliac crest bone autograft versus iliac crest bone allograft in the treatment of anterior shoulder instability with glenoid bone loss.

## Discussion

A trend exists toward increased utilization of bone-block stabilization for the treatment of shoulder instability among recently trained orthopedic surgeons [30]. With the better understanding of the role of “engaging” Hill-Sachs lesions in glenohumeral biomechanics and the specific indications for bone-block glenoid reconstruction, in combination with the use of meticulous preoperative planning, advanced imaging (3D reconstruction) of the glenohumeral bone defects, minimally invasive surgical techniques, sophisticated implants, and individualized evidence-based rehabilitation protocols, glenoid bone block augmentation surgery has rapidly evolved over the last few years [30]. Although a number of studies have been recently published in the literature, none of the previous reviews examined in a systematic manner the outcomes of contemporary-only iliac crest bone block techniques [17, 31, 32]. To address this point, we conducted a systematic review of contemporary literature including publications from the last 12 years.

The most important finding of this analysis was that, regardless of the fixation method, iliac crest bone block grafting was a satisfactory treatment for cases with recurrent anterior instability and substantial glenoid bone loss, since it resulted in low all-cause reoperation (6.1%) rate. The rates of recurrent instability (4.8%) and positive anterior apprehension test (4.8%) were also very low in the short term. Furthermore, regardless of the graft type (bicortical or tricortical autograft, J-shaped autograft, allograft), non-union (2.2%), osteolysis of the graft (0.4%), graft fracture (0.9%), or infection (1.7%) were very rarely noticed. Finally, hardware-related complications, such as screw breakage or symptomatic mechanical irritation around the screw insertion, were not common (3.9%). Based on these findings, we suggest that the contemporary use of iliac crest bone grafting is safe and effective in the short term for the management of anterior shoulder instability with substantial glenoid bone deficiency.

Whereas historical goals centered on the stable reduction and prevention of recurrent dislocation, current standards of success are predicated on the restoration of motion and strength and the return to functional activities, including competitive athletics [33]. In our analysis, the use of iliac crest bone graft resulted in significantly improved functional scores after surgery. In addition, postoperative ROM was significantly improved with none to minimal rotational loss. Finally, it was shown that the majority of patients who were treated with iliac crest bone grafting returned to their pre-injury level of athletic activities [21, 22, 28].

Recurrent glenohumeral instability represents a treatment challenge for orthopedic surgeons as it not only has the potential to result in a subsequent surgery, therapy, and missed activity time, but also has been associated in the past with long-term degenerative joint changes [34]. Although none of the studies included in our analysis showed any progression of osteoarthritis after short- to mid-term follow-up, we did not find any study to examine this variable in the long term. Based on this finding, it could be supported that contemporary iliac crest bone block techniques are not associated with short-term degenerative joint changes, but further studies are required to assess its long-term effect.

Iliac crest bone blocks that have been used for glenoid reconstruction were either autografts or allografts. Although the iliac crest is a convenient source of customizable autologous bone grafts, it has been associated with a substantial risk of chronic degenerative changes in the glenohumeral joint as well as immediate and, sometimes, persistent pain at the donor site [35, 36]. Allogeneic osteochondral iliac crest grafts were introduced to minimize the risk of arthropathy and donor site morbidity [18]. Although concerns have been raised regarding potential early resorption and inadequate osseointegration of the graft, an allograft-focused review showed that allograft reconstruction for glenoid bone loss provided excellent clinical outcomes, low rates of recurrent instability, and high osseous incorporation rates with no evidence of graft resorption [37].

In our analysis, both iliac crest autograft and allograft resulted in excellent survival rates and high osseous incorporation, although there was no study to directly compare them. Neither allograft nor autograft resorption occurred in the patient cohorts of our review. Moreover, no cases of osteoarthritic progression were found either with the use of autograft or allograft. Problems related to the donor site of the autograft, such as hypoesthesia, wound-related complications, or persistent pain, were very rare. Taking into consideration these findings, we feel that further research of higher quality should be done to lead to definite conclusions regarding the use of iliac crest allografts for cases requiring glenoid reconstruction, when iliac crest autografts of good quality are available.

There was only one study [26] to compare arthroscopic versus open iliac crest bone grafting procedures reporting that the open group was associated with increased coverage of the glenoid defect (95% in the arthroscopic group vs. 98% in the open group), steeper mean impaction angle, and increased mean medial offset compared to the arthroscopic group. However, the clinical relevance of these radiographic findings was unclear, since the clinical outcomes of both techniques were excellent.

Furthermore, there was only one study to compare open iliac crest bone grafting and Latarjet technique at 2 years follow-up [24]. In this bicentric prospective randomized study of 60 patients with anterior shoulder instability and glenoid bone loss, Moroder et al. [24] found no significant differences in failure rate, ROM, functional scores, satisfaction, and strength between the two procedures. However, further studies are required to confirm that the outcome of iliac crest bone grafting does not significantly differ from the glenoid transfer.

This review was not without limitations. Most of the studies included in this analysis were single series of patients without any control group. All apart from one study [24] were of low level of evidence (either III or IV) with selection, detection, and performance bias that might have affected the validity of the outcomes reported. In addition, all except for three studies [24, 27, 29] (33.3%) declared that some of their authors had relevant conflict of interests. The “quality assessment” of the studies for methodological deficiencies, as a common alternative to “risk of bias,” was examined by the modified Coleman methodology score [20], and it was to be found low to moderate. The study design, including the type of graft, type of fixation, follow-up, and type of surgery (open or arthroscopic), was relatively heterogeneous. In addition, there was a complete lack of mid- or long-term results. However, all studies examined a specific surgical technique (iliac crest bone block), which has gained increasing attention among physicians in recent years (almost all publications were from 2014 to 2018). In addition, the results of all studies were towards the same direction, since they all depicted that iliac crest bone block resulted in satisfactory clinical outcomes with low failure rates.

## Conclusions

Iliac crest bone block techniques in contemporary practice are safe and effective in the short-term (< 4 years) follow-up for the management of anterior shoulder instability with substantial glenoid bone deficiency. However, further studies of higher quality and longer follow-up are required to establish the therapeutic value of these techniques as well as to clarify whether there are differences in the outcomes of arthroscopic and open iliac crest bone block procedures.

## Data Availability

Data sharing not applicable to this article as no datasets were generated or analyzed during the current study.

## Abbreviations

ASOSS: Athletic Shoulder Outcome Scoring System
COI: Conflict of interest
F: Females
FU: Follow-up
LOE: Level of evidence
M: Males
m: Months
MCS: Modified Coleman Score
MISS: Melbourne Instability Shoulder Score
Not rep.: Not reported
PRISMA: Preferred Reporting Items for Systematic reviews and Meta-analyses
ROM: Range of motion
SSV: Subjective shoulder value
TW: Text word
VAS: Visual analog scale
WOSI: Western Ontario Shoulder Instability Index
y: Years
